# Melatonin promotes neuroblastoma cell differentiation by activating hyaluronan synthase 3‐induced mitophagy

**DOI:** 10.1002/cam4.2389

**Published:** 2019-07-05

**Authors:** Wen‐Jui Lee, Li‐Ching Chen, Juo‐Han Lin, Tzu‐Chun Cheng, Ching‐Chuan Kuo, Chih‐Hsiung Wu, Hui‐Wen Chang, Shih‐Hsin Tu, Yuan‐Soon Ho

**Affiliations:** ^1^ Ph.D. Program for Neural Regenerative Medicine, College of Medical Science and Technology Taipei Medical University and National Health Research Institutes Taipei Taiwan; ^2^ Division of Breast Surgery, Department of Surgery Taipei Medical University Hospital Taipei Taiwan; ^3^ Taipei Cancer Center Taipei Medical University Taipei Taiwan; ^4^ TMU Research Center of Cancer Translational Medicine Taipei Medical University Taipei Taiwan; ^5^ Ph.D. Program for Cancer Molecular Biology and Drug Discovery, College of Medical Science and Technology Taipei Medical University and Academia Sinica Taipei Taiwan; ^6^ School of Medical Laboratory Science and Biotechnology, College of Medical Science and Technology Taipei Medical University Taipei Taiwan; ^7^ Institute of Biotechnology and Pharmaceutical Research National Health Research Institutes Zhunan Taiwan; ^8^ Department of Surgery En Chun Kong Hospital New Taipei City Taiwan; ^9^ Department of Surgery, School of Medicine, College of Medicine Taipei Medical University Taipei Taiwan; ^10^ Department of Laboratory Medicine Taipei Medical University Hospital Taipei Taiwan; ^11^ Graduate Institute of Neural Regenerative Medicine, College of Medical Science and Technology Taipei Medical University Taipei Taiwan; ^12^ Graduate Institute of Medical Sciences, College of Medicine Taipei Medical University Taipei Taiwan

**Keywords:** autophagy, hyaluronan synthase 3, melatonin, N2a cell differentiation, neuroblastoma

## Abstract

Neuroblastoma is the second most common pediatric malignancy and has a high rate of spontaneous remission. Uncovering the mechanisms underlying neuroblastoma cell differentiation is critical for therapeutic purposes. A neuroblastoma cell line (N2a) treated with either serum withdrawal (<2.5%) or melatonin (>0.1 nmol/L) for 24 hours was used as a cell differentiation research model. Interestingly, the hyaluronan synthase 3 (HAS3) protein was induced in differentiated N2a cells. N2a‐allografted nude mice received an intraperitoneal injection of melatonin (40 or 80 mg/kg/day for 3 weeks). The mean tumor volume in mice treated with 80 mg/kg melatonin was smaller than that in PBS‐treated mice (1416.3 and 3041.3 mm^3^, respectively, difference = 1625 mm^3^, *P = 0.0003, n = 7 per group). Compared with the vector control group, N2a cells with forced HAS3 overexpression showed significantly increased neuron length (*P = 0.00082) and neurite outgrowth (*P = 0.00059). Intracellular changes in autophagy, including distorted mitochondria with abnormal circular inner membranes, were detected by transmission electron microscopy (TEM). Our study demonstrated that HAS3‐mediated signaling activated by physiological concentrations of melatonin (>0.1 nmol/L) triggered significant N2a cell differentiation. These results provide molecular data with potential clinical relevance for therapeutic drug development.

## 1 INTRODUCTION

Peripheral neuroblastic tumors compose a family of common solid tumors found in children.^1^, ^2^, ^3^ They are defined as neuroblastomas, ganglioneuroblastomas or ganglioneuromas depending on the level of neuroblastic differentiation, the content of the stromal cells, and other specific features.^4^ Neuroblastoma is a common malignant childhood tumor of the sympathetic nervous system, accounting for up to 10% of pediatric cancers and 15% of cancer‐related pediatric deaths.^5^ Histologically, neuroblastomas range from tumors containing poorly differentiated neuroblasts to those composed of fully differentiated sympathetic neurons. Patients with poorly differentiated neuroblastomas upon histological examination have significantly poorer survival than patients with well‐differentiated neuroblastomas. The prognosis is poor for patients with undifferentiated neuroblastoma subtypes, with a 5‐year overall survival of only 50%, while the 5‐year overall survival improves slightly to 69% for patients with poorly differentiated tumors, representing an intermediate prognosis.^6^ However, the genes involved in driving the differentiation of neuroblastoma cells remain uncertain.

Melatonin (*N*‐acetyl‐5‐methoxytriptamine) is an endocrine hormone released primarily by the pineal gland.^7^, ^8^, ^9^ Melatonin can easily penetrate the blood‐brain barrier, which allows it to control multiple physiological functions, such as insulin production and secretion, circadian rhythm, stress, mood, sleep, and sexual maturation.^10^, ^11^ Although melatonin is well known as an endogenous circadian clock regulator, it also reduces drug‐induced free radical generation and exerts neuroprotective effects.^12^, ^13^, ^14^ A recent study found that melatonin enhances the oligodendrocyte differentiation of murine cortical neural stem cells.^15^ These results suggest that the melatonin‐induced differentiation of undifferentiated neuroblastoma cells might represent an important process with potential therapeutic benefits.

Macroautophagy/autophagy comprise several processes by which organelles are degraded via recycling in the lysosome. These organelles include mitochondria, the selective degradation of which is known as mitophagy. Mitophagy begins with the formation of double‐membraned, autophagosome‐engulfed mitochondria. A recent study demonstrated that treating head and neck cancer with a combination of melatonin and rapamycin significantly activated mitophagy by regulating mitochondrial function. Another study indicated that melatonin specifically induces cancer cell differentiation.^16^ However, for normal cells, melatonin supplementation can robustly reduce damage to liver and mitochondrial function and protect from the pathogenesis of nonalcoholic fatty liver disease.^17^ Collectively, these studies suggest that melatonin can be used as an adjuvant with chemotherapeutic agents (such as rapamycin), improving effectiveness while minimizing side effects. According to previous studies, which demonstrated that autophagic proteins increase during neuronal differentiation of fetal neuro stem progenitor cells (NSPCs). For instance, the scientist found that the Atg9a levels and the LC3‐II/LC3‐I ratio raised during neurogenesis in the NSPCs derived from the forebrain.^18^ Similarly, another studies also demonstrated that the Atg5, Eva1a, and LC3II proteins were detected in a high level in the mouse cerebral cortex during the neurogenic process.^19^, ^20^ In contrast, inhibition of the Atg5 by siRNA impaired cortical neuronal differentiation while increasing proliferation of NSPCs.^20^ Such studies implied that induction of autophagy may play an important role in N2a cells differentiation.

CD44 is the major glycoprotein receptor for hyaluronan (HA), a component of cell matrices, and most of its known functions are attributed to its ability to recognize HA. Surprisingly, previous papers have demonstrated that CD44 negative (CD44^‐^) neuroblastoma cells are associated with all the phenotypic and molecular features required for neuroblastoma‐initiating cell growth.^21^, ^22^ However, in this study, we found that melatonin induced the expression of the hyaluronan synthase 3 (HAS3) protein. In the normal brain, the HAS3‐generated HA protein accumulates in the extracellular matrix. Additionally, HA impedes neuroblastoma (HTLA230) cells from inducing cancer cell differentiation.^23^ These results can explain why initial CD44^‐^ neuroblastoma cells^21^, ^22^ can grow by escaping from the neuronal extracellular matrix, with its high levels of HA. These results imply that one physiological function of melatonin‐induced HAS3 expression is to diminish the initiation of neuroblastoma cell formation via the binding of HA to the CD44 receptor and the induction of cancer cell differentiation.

To test this hypothesis, we established a cell differentiation model using physiological concentrations of melatonin to treat N2a neuroblastoma cells. Melatonin significantly induced HAS3 and the expression of related cellular differentiation markers. The forced expression of HAS3 triggered neurite outgrowth in differentiated cells. Our study provides a molecular basis for understanding the mechanisms by which melatonin prevents the initiation of neuroblastoma cell growth via the activation of HAS3‐mediated cellular differentiation. These results may be important for clinical diagnosis and be valuable for the design of therapeutic drugs for cancer differentiation.

## 2 MATERIALS AND METHODS

### 2.1 Cell culture

The mouse neuroblastoma cell line N2a is generally studied for neuronal differentiation research in vitro.^24^, ^25^ N2a cells were cultured in Dulbecco's Modified Eagle Medium (DMEM, Gibco 12100046, CA) supplemented with 10% heat‐inactivated fetal bovine serum (FBS; Gibco A38401, CA), 50 U/mL penicillin/streptomycin/neomycin (Invitrogen 15640055, CA), nonessential amino acid solution (Thermo Fisher Scientific 11140050, CA) and sodium pyruvate (Sigma Aldrich P5280, MI) in a humidified (5% CO_2_, 37°C) incubator.

### 2.2 Cell counting

N2a cells were seeded onto 10‐cm plates to achieve 10^5^ cells at 24 hours. The cells were treated with 0, 0.1, 1, and 10 nmol/L of melatonin for 24 hours. After trypsin (Thermo Fisher Scientific R001100, CA) process, 10 μL of medium content cells was mixed with 10 μL of trypan blue (Thermo Fisher Scientific T10282, CA) and counted by microscopy (Leica, Wetzlar, Germany).

### 2.3 Cell differentiation assay

To establish the N2a cell differentiation model, the cells were seeded onto 10‐cm plates to achieve 10^5^ cells at 24 hours. The cells were treated with either melatonin (Sigma Aldrich M5250, St. Louis, MI) (0.1, 1, and 10 nmol/L for 24 hours) or serum deprivation (0%‐10% FBS, for 24 hours) and transfected with an HAS3 overexpression plasmid. The differentiated cells were detected by immunofluorescence staining to evaluate differentiation markers such as βIII tubulin (TUBB3; GeneTex GTX631836, CA) and GFAP (GeneTex GTX108711, CA). To indicate the total length of neurite extension in the melatonin‐induced differentiated N2a cells, we randomly selected six microscopic fields (1000 cells counted per field) in a 10‐cm plate. The length of neurite extension in each cells were measured (using ImageJ software, NIH, USA). The measured length of neurite results from all these cells were sum from six fields and averaged number per field is indicated at the Y‐axis. At least three experiments were performed for each stimulation condition and their mean value was calculated. To assess differentiation, cells bearing neurite processes 1.5 times longer than their cell bodies were considered differentiated.

### 2.4 Flow cytometry

To investigate the cell cytotoxicity after melatonin treatment, N2a cells were cultured above 80% confluent and then synchronized in Dulbecco's Modified Eagle Medium (DMEM, Gibco 12100046, CA) supplemented with 0.04% of heat‐inactivated fetal bovine serum (FBS; Gibco A38401, CA). After synchronization, the medium was replaced with 10% of FBS and 10 nmol/L of melatonin for 24 hours. Cells were fixed with 70% of cold ethanol for 1 hour and then added with RNaseA (Sigma Aldrich 10109169001, St. Louis, MI) in 37°C water bath for 30 minutes. After RNaseA treatment, all the samples were stained with propidium iodide (PI) (Sigma Aldrich P4170, St. Louis, MI) at room temperature for 15 minutes. About 10 000 cells of each sample were detected by FACS Calibur flow cytometry (BD, NJ).

### 2.5 Immunofluorescence and confocal microscopy

Cells were fixed with 4% paraformaldehyde (Sigma Aldrich 158127, St. Louis, MI) in PBS for 15 minutes at room temperature and permeabilized with 0.1% Triton X‐100 (Sigma Aldrich X100, St. Louis, MI) in PBS for 5 minutes at room temperature. Samples were blocked for 30 minutes in PBS with 2% bovine serum albumin (BSA, Sigma Aldrich A2153, St. Louis, MI). Primary antibodies against *β*‐tubulin (GeneTex GTX11307, CA), HAS3 (Sigma Aldrich SAB2108148, St. Louis, MI), GFAP (GeneTex GTX108711, CA) and TUBB3 (GeneTex GTX631836, CA) were diluted 1:100 in PBS with 1% BSA and then incubated for 2 hours at room temperature. AffiniPure goat anti‐mouse‐FITC (Jackson ImmunoResearch 115‐095‐062, PA) and AffiniPure goat anti‐rabbit rhodamine (Jackson ImmunoResearch 111‐025‐003, PA) secondary antibodies were diluted 1:50 and incubated for 1 hour at room temperature. Coverslips were mounted with VECTASHIELD Antifade Mounting Medium (Vector Laboratories H‐1000, CA). FRET activity assay was measured by Leica FRET AB system and all the images were captured by confocal microscopy (Leica, Wetzlar, Germany).

### 2.6 Transfection and electroporation of cells

HAS3 overexpression vector which constructed in pcDNA3.1 + plasmid (Invitrogen V79020, CA) with fusion protein EYFP and HAS3 siRNA which sequence was TGGGCCTGCACCTGCTCAT constructed in pSUPER (Oligoengine VEC‐pBS‐0006, WA) vector were transfected into N2a cells via electroporation using 1100 V, 30 milliseconds, 2 times. The electroporation mixture contained 5 × 10^6^ cells with 10 μg of plasmid. The efficiency of transfection was determined by fluorescence microscopy (Leica, Wetzlar, Germany) and confocal microscopy (Leica, Wetzlar, Germany).

### 2.7 Protein extraction and western blotting

For protein extraction, cells were washed twice with ice‐cold PBS and lysed on ice in golden lysis buffer (20 mmol/L Tris‐HCl (Sigma Aldrich RES3098T‐B7, St. Louis, MI), pH 8.0, 137 mmol/L NaCl (Sigma Aldrich S7653, St. Louis, MI), 5.95 mmol/L EDTA (Sigma Aldrich 1233508, St. Louis, MI), 5 mmol/L EGTA (Sigma Aldrich E3889, St. Louis, MI), 10 mmol/L NaF (Sigma Aldrich S7920, St. Louis, MI), 1% Triton X‐100 (Sigma Aldrich X100, St. Louis, MI), and 10% glycerol (Sigma Aldrich G5516, St. Louis, MI)) supplemented with protease inhibitors (Sigma Aldrich P8340, St. Louis, MI) and phosphatase inhibitors (Sigma Aldrich P2850, St. Louis, MI). The proteins were separated via 12% sodium dodecyl sulfate‐polyacrylamide gel electrophoresis (SDS‐PAGE) and transferred to PVDF membranes (Thermo Fisher Scientific LC2002, CA). HAS3 (Sigma Aldrich SAB2108148, St. Louis, MI), GFAP (GeneTex GTX108711, CA), *β*‐tubulin (GeneTex GTX11307, CA), TUBB3 (GeneTex GTX631836, CA) and *α*‐actin (GeneTex GTX109639, CA) antibodies were diluted 1:2000 in TBST, and the membranes were incubated for 2 hours at room temperature. Horseradish peroxidase (HRP)‐conjugated anti‐mouse and anti‐rabbit IgG (Santa Cruz Biotechnology SC‐2005, CA) secondary antibodies were diluted 1:4000, and the membranes were incubated for 1 hour at room temperature. *α*‐Actin was used as the protein loading control.

### 2.8 Immunohistochemistry (IHC) analysis

Fixed, frozen tissues were sectioned at a thickness of 7 μm. Frozen sections were fixed with methanol for 15 minutes. All slides were immersed in 3% hydrogen peroxide (H_2_O_2_) (Sigma Aldrich 21673, St. Louis, MI) for 5 minutes to block endogenous peroxidase activity. The slides were incubated in 0.1% BSA blocking buffer for 15 minutes. The slides were incubated with primary antibody (1:100 dilution) for an hour. The slides were incubated with the secondary antibody for another 30 minutes. Then, the signals were detected and amplified using a biotinylated streptavidin‐HRP and 3,3′‐diaminobenzidine tetrahydrochloride (DAB) system (Dako Corp K3468, CA). Tissue specimens were stained with hematoxylin to locate the nuclei. All slides were dehydrated in an alcohol gradient and covered with coverslips and mounting medium.

### 2.9 Acridine orange (AO) staining

The acidic compartments of the cells were labeled with AO (Thermo Fisher Scientific A1301, CA) as a marker of autophagy. N2a cells were incubated with AO (1 μg/mL in PBS) for 15 minutes and rinsed with PBS three times at 37°C in the dark. Cells were imaged using a fluorescence microscope (Leica, Wetzlar, Germany).

### 2.10 Transmission electron microscopy (TEM)

After HAS3 transfection, N2a cells were seeded on chamber slides for 24 hours. Samples were washed with 0.1 mol/L cacodylate (Sigma Aldrich C0250, St. Louis, MI) buffer in artificial seawater (ASW) (Sigma Aldrich 95577, St. Louis, MI) and fixed in 0.1 mol/L cacodylate buffer in ASW containing 2.5% glutaraldehyde (Sigma Aldrich G7776, St. Louis, MI) in ASW for 1 hour at room temperature. The cells were postfixed in 1% osmium tetroxide (Sigma Aldrich 419494, St. Louis, MI) in ASW for 90 minutes. Subsequently, the samples were dehydrated through a graded ethanol (Sigma Aldrich E7023, St. Louis, MI) series and embedded in epoxy resin (Sigma Aldrich 755877, St. Louis, MI) overnight at 60°C. Images were acquired via TEM (Hitachi, Tokyo, Japan).

### 2.11 Animal experiments

Ethical approval of animal care and experiments was obtained from the Taipei Medical University Laboratory Animal Center (IACUC approval no: LAC‐2014‐0282). Specific pathogen‐free athymic (T‐cell‐deficient) nude mice were obtained from the National Laboratory Animal Center (NARLabs, Taipei, Taiwan). Suspensions of 10^6^ cells in 50 μL of PBS were injected into the dorsal subcutis of the mice (n = 24) using sterile syringes. Subsequently, the mice were divided into three groups: (a) the control group, which received PBS; (b) received 40 mg/kg melatonin; (c) received 80 mg/kg melatonin. The tumors were measured every 7 days using an IVIS 200 imaging system (Xenogen, CA) and calipers until the animals were sacrificed.

### 2.12 Preparation of protein isolates from N2a‐cell‐based tumor tissues

N2a‐cell‐based tumors were cut into 5‐mm^3^ cubes. All tumors were placed in 2‐mL Eppendorf tubes with a small steel ball and then homogenized with an SH‐100 homogenizer (J & H Technology, Taipei, Taiwan). Lysed tissues were mixed with golden lysis buffer (20 mmol/L Tris‐HCl (Sigma Aldrich RES3098T‐B7, St. Louis, MI), pH 8.0, 137 mmol/L NaCl (Sigma Aldrich S7653, St. Louis, MI), 5.95 mmol/L EDTA (Sigma Aldrich 1233508, St. Louis, MI), 5 mmol/L EGTA (Sigma Aldrich E3889, St. Louis, MI), 10 mmol/L NaF (Sigma Aldrich S7920, St. Louis, MI), 1% Triton X‐100 (Sigma Aldrich X100, St. Louis, MI), and 10% glycerol (Sigma Aldrich G5516, St. Louis, MI)) supplemented with protease inhibitors (Sigma Aldrich P8340, St. Louis, MI) and phosphatase inhibitors (Sigma Aldrich P2850, St. Louis, MI). The mixtures were centrifuged at 12 000 rpm and 4°C. The supernatants were collected for Western blot assays.

### 2.13 Statistical analysis

SPSS version 21.0 for Windows (IBM) was used for all statistical analyses. Neurite outgrowth was evaluated by counting 1000 cells per condition. Neurite number and length were quantified using ImageJ Pro‐Plus software. In each analysis, the data represent the mean ± SEM of at least three independent experiments. For comparison, statistical significance was tested using *t *tests. All *P* values were based on two‐sided statistical analyses, and *P* < 0.05 was considered statistically significant.

## 3 RESULTS

### 3.1 Serum deprivation induces neuroblastoma (N2a) cell differentiation by inducing HAS3 protein

**Figure 1:**
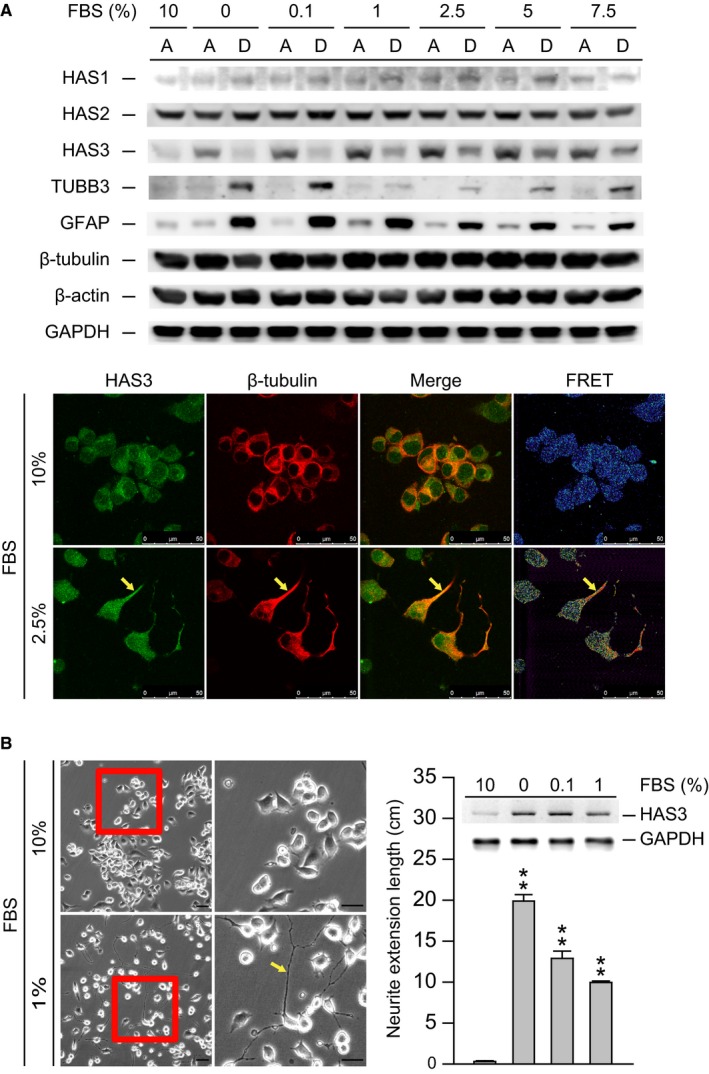
Starvation induces N2a cell differentiation via HAS3 upregulation. (A) N2a cells were treated with 0%‐10% serum for 24 h. The treated cells were assessed for HAS1, HAS2, HAS3, TUBB3, GFAP, *β*‐tubulin, and *α*‐actin by Western blot analysis (A = attached cells, D = detached cells). The results were normalized to *α*‐actin. N2a cells were treated with 2.5 or 10% serum. Differentiated cells were stained with HAS3‐rhodamine and *β*‐tubulin ‐FITC. The colocalization of HAS3 and *β*‐tubulin was measured by FRET analysis. Magnification, 630×; scale bar, 50 μm. (B) N2a cells were treated with 0%, 0.1%, 1% or 10% serum. The morphology of the differentiated cells was captured by microscopy. Magnification, 100×; scale bar, 10 μm. Differentiated cells were assessed for HAS3 and GAPDH by Western blot analysis. The results were normalized to GAPDH. Neurite length was measured in six randomly selected microscopic fields using ImageJ software. The data are presented as the mean ± SD;    **P < 0.01 compared with the control group

A previous paper demonstrated that HA induced the differentiation of CD44‐positive (CD44+) human neuroblastoma (HTLA230) cells.^23^ These results implied that CD44 + neuroblastoma cells are sensitive to HA‐induced differentiation, which explains why CD44^‐^ cells are detected preferentially in neuroblastoma patients.^21^, ^22^ To explore the mechanisms of HA‐induced neuroblastoma cell differentiation, mouse N2a cells were serum deprived (0.1%‐7.5%) for 24 hours to establish an in vitro differentiation model. The differentiated (attached, denoted A) and undifferentiated (detached, denoted D) cells were harvested separately for immunoblot analysis (Figure 1A). The protein levels of three different HA synthases (HAS1, 2, and 3) in the serum‐deprived N2a cells were determined. Interestingly, we found that compared with HAS1 and HAS2, HAS3 were induced significantly in the differentiated N2a cells (Figure 1A). The TUBB3 and GFAP proteins were analyzed as molecular markers of differentiation and were induced in the low serum (<2.5%, 24 hours)‐treated groups.^26^ A previous study demonstrated that retinoic acid (RA) treatment significantly upregulated the accumulation of the membrane protein GDE2 at the growth cones of neuroblastoma N2a cells during neuronal differentiation.^27^ Another in vivo study demonstrated that *β*‐tubulin, which is involved in neuronal differentiation, was located at the growth cones of N2a neurites.^28^ In this study, we performed fluorescence resonance energy transfer (FRET) assays in N2a cells and found that HAS3 interacted with *β*‐tubulin at the growth cones of differentiated N2a cells (Figure 1A, yellow arrow). The neuron length also increased in the serum‐deprived (<0.1% FBS) N2a cells in which HAS3 protein expression had been upregulated (Figure 1B). Collectively, these results implied that HAS3 protein expression is involved in the process of serum deprivation‐induced N2a cell differentiation.

### 3.2 Overexpression of HAS3 induces N2a cell differentiation

**Figure 2:**
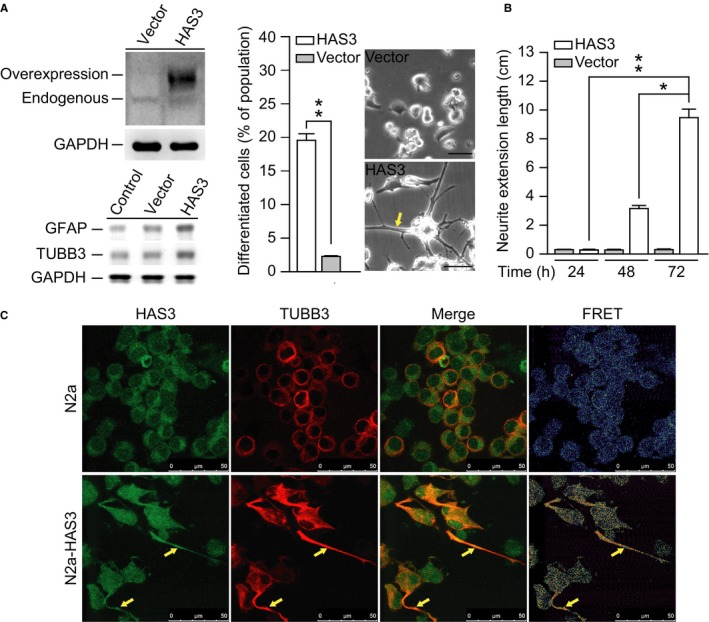
Overexpressing HAS3 in the N2a cells significantly promotes cell differentiation. (A) HAS3 overexpression plasmid was transfected into the N2a cells. The protein level of HAS3 and differentiation markers (TUBB3 and GFAP) were detected by Western blot assay (left panel). Differentiated N2a cells were calculated based on 1000 random cells in the vector control group and the HAS3 overexpression group. The morphological change after HAS3 overexpression was captured by microscopy. Magnification, 100×; scale bar, 10 μm. (B) Neurite length was measured in six random microscopic fields for both the vector control group and the HAS3 overexpression group at 24, 48 and 72 h. (C) Differentiated N2a cells were identified by immunofluorescence staining after transfection with an HAS3 expression plasmid. TUBB3‐rhodamine was used as a marker for differentiated N2a cells. Endogenous and overexpressed HAS3 were identified via HAS3‐FITC staining. Magnification, 630×; scale bar, 50 μm. The data are presented as the mean ± SD; *P < 0.05 and **P < 0.01

To investigate whether HAS3 was involved in N2a cell differentiation, we transfected undifferentiated N2a cells with an HAS3 overexpression plasmid. The differentiation markers were detected by Western blot assay and the results indicated that high level GFAP and TUBB3 proteins were detected in the HAS3‐induced differentiated N2a cells (Figure 2A).

The forced overexpression of the HAS3 protein significantly increased the number of differentiated N2a cells relative to that in the vector control group (Figure 2A, left, *P* = 0.000059). Compared to the vector controls, these differentiated N2a cells showed protruding neurite formation (Figure 2A, right, yellow arrow). Statistical analysis revealed that the neurons were significantly longer in the HAS3‐overexpressing cells than in the control group and that this difference was time‐dependent (Figure 2B, *P* = 0.000082). FRET analysis showed that HAS3 interacted with TUBB3, which is a neuroblastoma cell‐specific marker of differentiation (Figure 2C).^29^ These results demonstrate that overexpressing HAS3 either by serum deprivation (Figure 1) or by transfection with an HAS3 plasmid (Figure 2) triggers significant differentiation in N2a cells, as evidenced by increases in neuron length and related markers of differentiation.

### 3.3 Melatonin‐induced HAS3 expression promotes N2a cell differentiation

**Figure 3:**
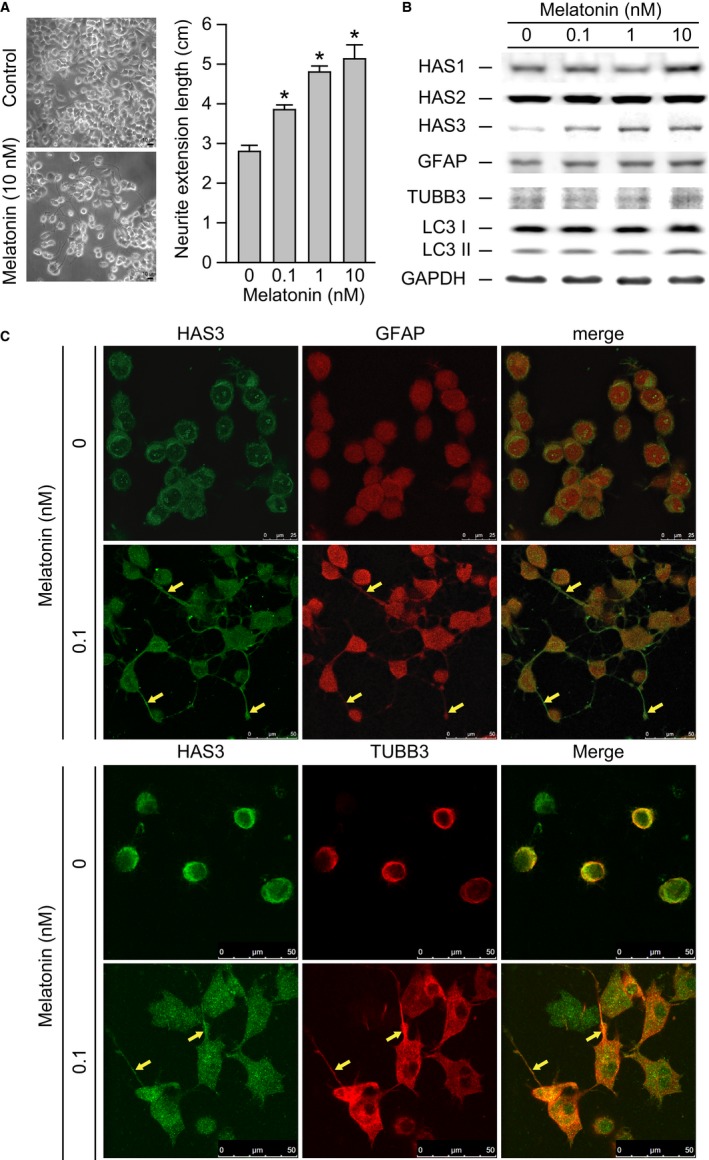
Melatonin promotes the neuronal differentiation of N2a cells. (A) The N2a cells gross morphology with or without 10 nmol/L melatonin treatment was presented (left). Magnification, 100×; scale bar, 10 μm. Neurite extension length was measured in six random microscopic fields after 0, 0.1, 1, or 10 nmol/L melatonin treatment. (B) Melatonin‐treated cells were assessed for HAS1, HAS2, HAS3, GFAP, TUBB3, LC3, and GAPDH expression using Western blot analysis. GFAP and TUBB3 were selected as differentiation markers. (C) Differentiated N2a cells were identified via immunofluorescence staining of GFAP‐, TUBB3‐rhodamine, and HAS3‐FITC after treatment with 0 or 0.1 nmol/L melatonin. Magnification, 630×; scale bar, 25 μm. The data are presented as the mean ± SD; *P < 0.05 compared with the control group

A previous study showed that melatonin promotes the differentiation of neural stem cells (NSCs) and neuronal cells.^30^ Another study demonstrated that melatonin enhanced the differentiation of induced pluripotent stem cells (iPSCs) into NSCs.^31^ To investigate whether melatonin exerts effects on N2a cell differentiation, N2a cells were treated with melatonin (0.1 to 10 nmol/L) for 48 hours, the N2a cell's dendrite protrusion was observed (Figure 3A, left) and the neuronal lengths of the differentiated N2a cells were measured (Figure 3A, right). Statistical analysis revealed that neuron length increased significantly and in a dose‐dependent manner after melatonin treatment compared with the control treatment (Figure 3A, *P* = 0.00045). Western blot assay showed that the HAS3 and GFAP proteins were upregulated in the N2a cells treated with the low dose (>0.1 nmol/L) of melatonin (Figure 3B). Interestingly, we found increased HAS1 expression in the N2a cells treated with a high concentration (>10 nmol/L) of melatonin (Figure 3B). The autophagy marker was also detected by Western blot assay in the melatonin (>10 nmol/L)‐induced differentiated N2a cells in which the LC3II protein level expression increased (Figure 3B). Immunofluorescence staining showed that the differentiation markers including HAS3, GFAP, and TUBB3 proteins were detected specifically on the plasma membrane protrusions of melatonin‐treated (0.1 nmol/L, 48 hours) N2a cells (Figure 3C, yellow arrow). These data demonstrate that melatonin‐induced HAS3 expression promotes N2a cell differentiation.

### 3.4 Autophagy detected in melatonin‐treated N2a cells

According to a previous study, melatonin‐induced autophagy can be detected in differentiated glioma‐initiating cells.^32^ In this study, we suggest that HAS3 overexpression promotes the differentiation of N2a cells induced by physiological concentrations of melatonin (~10 nmol/L) and facilitates autophagy to eliminate neuroblastoma‐initiating cells in healthy individuals. HAS3 could thus be a potential biomarker and target of clinical therapeutic relevance. To confirm the autophagic effects underlying melatonin treatment, we first excluded the possibility of N2a cell cytotoxicity induced by melatonin (Figure S1A and S1B). To test whether melatonin treatment effectively induced the N2a cells proliferation or mainly cancer cells differentiation, the N2a cells were treated with melatonin (0.1‐ 10 nmol/L) and the MAPK signaling or cell cycle signaling proteins were determined by Western blot assay and the results are shown in supplementary Figure 2B. We found that the p38 protein level was decreased after 10 nmol/L of melatonin treatment. We also checked cell cycle signaling pathway related proteins such as p21, p27 and cyclin B. However, there was no difference between control‐ and melatonin‐treated groups.

**Figure 4:**
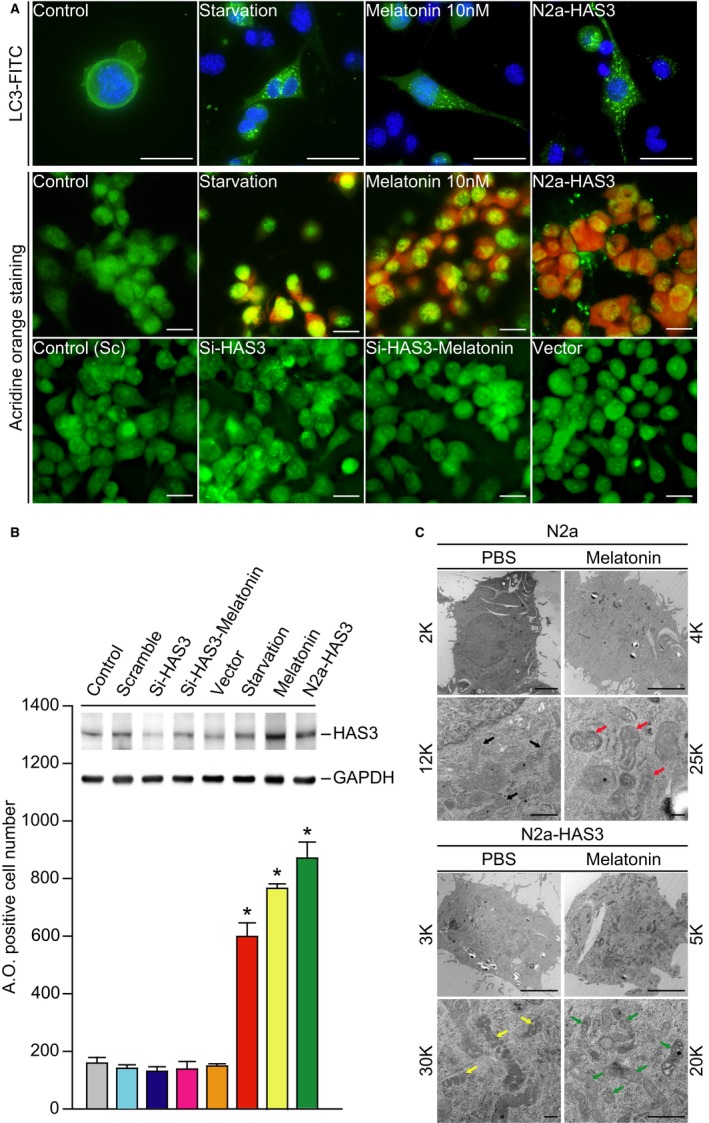
Melatonin induces N2a cell autophagy via HAS3 upregulation. (A) Fluorescence images of acidic vesicles stained with AO (1 µmol/L) and immunofluorescence staining results of LC3 protein expression following starvation, melatonin treatment (10 nmol/L) or HAS3 overexpression. Magnification, 630× and 100×; scale bar, 10 μm. (B) The quantitation of AO‐positive cells is presented for the starvation, melatonin (10 nmol/L) and HAS3 overexpression groups. Each group of cell lysates was assessed for HAS3 and GAPDH expression by Western blot analysis. (C) Mitochondrial damage was detected by TEM in melatonin‐treated and untreated HAS3‐induced N2a cells and in control N2a cells. The data are presented as the mean ± SD; *P < 0.05

Cell counting and flow cytometry analysis showed that there was no significant cytotoxicity after treatment with physiological levels of melatonin (10 nmol/L). We then performed AO and the LC3 immunofluorescence staining to observe the melatonin‐induced autophagic cells (Figure 4A). The results indicated that LC3 positive‐stain cells were significantly detected in the serum‐starved, melatonin‐treated, and HAS3‐overexpressing cells than in the controls (Figure 4A, upper panel). Similar results were also seen in the AO stain experiments (Figure 4A, middle panel, and 4B, bars 6‐8). Interestingly, siRNA knockdown of HAS3 protein expression significantly reversed the melatonin‐induced autophagic effects (Figure 4A, lower panel, and 4B, bar 4). Our results implied that HAS3 participates in melatonin‐induced autophagy in N2a cells. To test this hypothesis, N2a cells were transfected with the HAS3 plasmid and treated with melatonin (10 nmol/L, 24 hours) or left untreated, and cellular morphological changes were observed by TEM (Figure 4C). In the control N2a cells, normal mitochondria were observed. The inner mitochondrial membrane was compartmentalized into numerous cristae, which increase the surface area of the inner mitochondrial membrane, enhancing its ability to produce ATP (Figure 4C, black arrow). However, in the melatonin‐treated N2a cells, abnormal circular‐shaped cristae that contained the mitochondrial matrix were detected (Figure 4C, red arrow). These results were consistent with previous studies that demonstrated that melatonin enhances mitophagy in various diseases.^33^, ^34^, ^35^ Interestingly, abnormal crista formation was also detected in the N2a cells transfected with the HAS3 plasmid (Figure 4C, yellow arrow). We further found that compared with the other groups, exposing HAS3‐transfected N2a cells to melatonin (10 nmol/L, 24 hours) dramatically enhanced abnormal crista formation (Figure 4C, green arrow). These results suggested that HAS3 plays a critical role in initiating melatonin‐induced mitophagy in N2a cells.

### 3.5 Melatonin inhibits N2a‐allografted tumor growth in vivo

**Figure 5:**
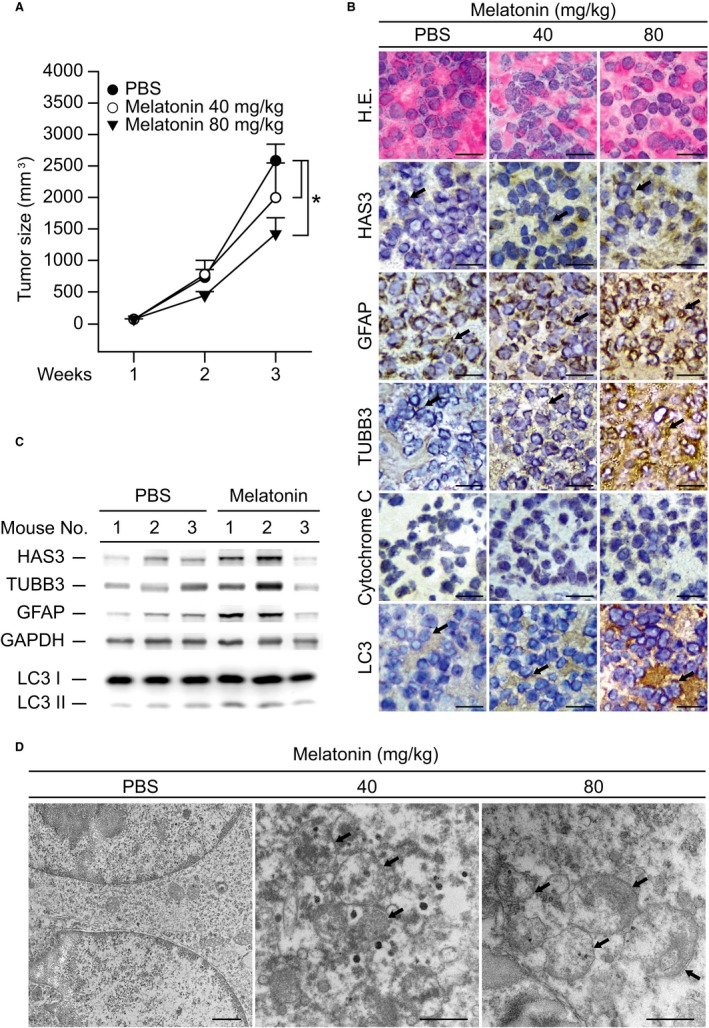
Melatonin inhibits tumor growth and promotes autophagy in vivo. (A) Tumor size was reduced by melatonin treatment. PBS, 40 mg/kg melatonin or 80 mg/kg melatonin was intraperitoneally injected into nude mice once a day for 3 weeks. The horizontal bars indicate the average tumor size. (B) Tumor sections from each group were analyzed for HAS3, GFAP, TUBB3, cytochrome C, and LC3 via IHC staining. (C) Each group of tumor lysates was assessed for HAS3, TUBB3, GFAP, LC3, and GAPDH. The results were normalized to GAPDH. (D) Autophagosomes were detected by TEM in tumor tissue from each group. Magnification, 12 K; scale bar, 1 μm. The data are presented as the mean ± SD; *P < 0.05

As described above, melatonin induced N2a cell differentiation by activating HAS3‐mediated mechanisms. To confirm this observation, an in vivo study was performed in N2a‐allografted nude mice treated with melatonin (40‐80 mg/kg) for 3 weeks (Figure 5A). The N2a‐allografted tumors grew rapidly in the PBS‐treated control mice. The N2a‐allografted tumor growth was inhibited significantly in the group treated with a high dose of melatonin (80 mg/kg) (Figure 5A, *P* = 0.003). To understand whether melatonin inhibited N2a‐allografted tumor growth via cell differentiation and autophagy, we investigated each tumor tissue using IHC, immunoblotting and TEM (Figure 5B‐D). Serial tumor tissue sections were taken, and HAS3, differentiation markers (GFAP and TUBB3), and an autophagic marker (LC3) were detected by IHC (Figure 5B). The IHC data demonstrated that HAS3 was induced by melatonin treatment in a dose‐dependent manner in N2a‐allografted tumor tissue. The differentiation (GFAP and TUBB3) and autophagy (LC3) markers were also induced in the melatonin‐treated tumors. On the other hand, an apoptosis‐related marker (cytochrome C) was not changed in the melatonin‐treated N2a tumors (Figure 5B). To validate the protein expression levels, we homogenized tissue from each tumor, and HAS3 and both differentiation markers in the N2a‐allografted tumors were analyzed via immunoblotting (Figure 5C). The protein expression levels of HAS3, GFAP, TUBB3, and LC3 increased in the melatonin‐treated N2a‐allografted tumors (Figure 5C). We then performed TEM assays using the N2a‐allografted tumors, and the results indicated that there were many more double‐membraned autophagosomes in the melatonin‐treated tumors than in the control (PBS‐treated) tumors (Figure 5D, black arrow).

**Figure 6:**
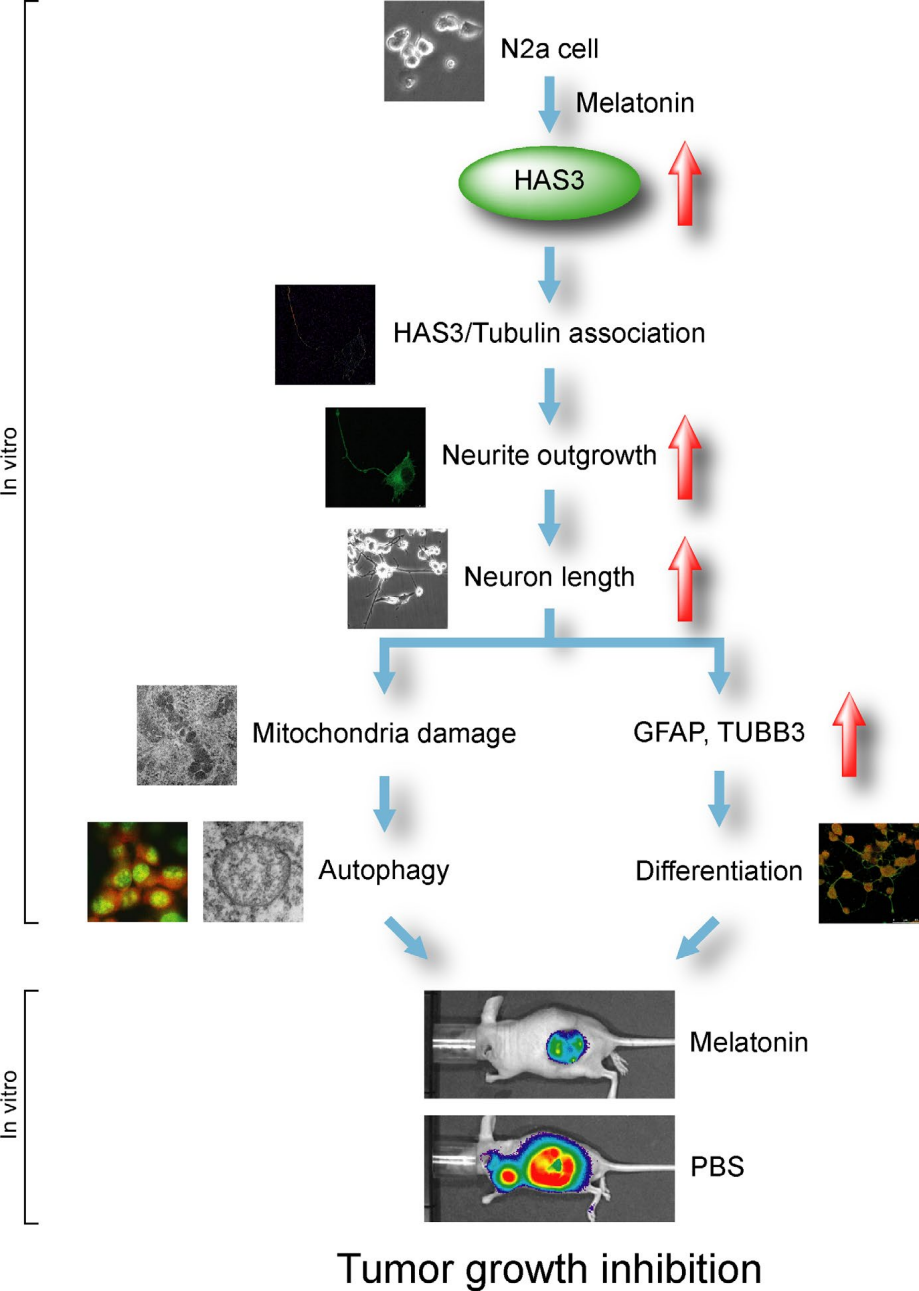
Model of melatonin treatment promoting N2a cell differentiation in vitro and inhibiting tumor growth in vivo

Together, these studies suggest that exposure to physiological levels (<10 nmol/L) of melatonin triggers N2a tumor cell differentiation which abolishes the initial step carcinogenesis. Western blot data revealed that the differentiation markers GFAP and TUBB3 were significantly increased in differentiated N2a cells. Overexpression of HAS3 significantly induced the N2a cell neurite outgrowth as well as increased the neuron length was detected. Autophagy was detected in the differentiated N2a cells by AO staining and TEM after melatonin treatment (Figure 6). These mechanisms prevent tumor progression by activating HAS3‐mediated effects, which then induce autophagy and eventually eliminate tumor cell growth in vivo.

## 4 DISCUSSION

Differentiation therapy is one way to control the growth of neuroblastoma cells by initiating differentiation and apoptosis.^36^ In this study, we demonstrated that serum starvation, as well as exposure to physiological levels (<10 nmol/L) of melatonin, induced N2a cell differentiation via the activation of HAS3‐mediated signals. A previous study also demonstrated that HAS3 overexpression markedly promotes filamentous actin aggregation in neuronal protrusions.^37^ The HA protein, which is generated by HAS3, significantly induces CD44 + neuroblastoma cell differentiation.^23^ However, the CD44^‐^ neuroblastoma cells escape melatonin‐induced HA‐mediated cell differentiation; they can then cause tumor formation.^21^, ^22^ These findings suggest that inducing HAS3 expression may be useful as a novel strategy for neuroblastoma cell differentiation therapy.

Previous studies indicate that sleep problems, including light exposure at night, night/shift work, late sleeping, and frequent night waking, could increase the risk of developing cancer.^38^, ^39^, ^40^, ^41^, ^42^, ^43^ Artificial light exposure at night (LAN)‐induced circadian disruptions promote cancer by inhibiting melatonin‐induced antitumor effects.^44^ A previous study demonstrated that cancer patients had lower levels of melatonin than the control group,^45^ and sleep deprivation (LAN) has been recognized by the World Health Organization (2007) as a probable carcinogen.^44^ Interestingly, a recent study demonstrated that physiological concentrations of melatonin inhibit the proliferation and invasiveness of human breast cancer cells.^46^ Our study further demonstrated that physiological levels (<10 nmol/L) of melatonin induced neuroblastoma cell differentiation. These results imply that melatonin is important for the blockade of early‐stage neuroblastoma cell proliferation by initiating cancer cell differentiation.

A previous study demonstrated that berberine, a plant alkaloid, induces neuronal differentiation accompanied by increases in differentiation markers such as MAP2, TUBB3 and NCAM in N2a cells.^47^ Additionally, TLR4 plays a key role in activating NF‐*κ*B‐mediated effects to trigger the differentiation of glioblastoma stem‐like cells.^48^, ^49^ HA (100 μg/mL) synthesized by HAS3 is a main component of the brain extracellular matrix, and effectively triggers the TLR4‐NF‐κB pathway in differentiating glioblastoma stem‐like cells.^50^ Melatonin (50 μmol/L‐1 mmol/L) combined with 5‐10 nmol/L vincristine or with 100 μmol/L‐1 mmol/L ifosfamide significantly increased caspase‐3, caspase‐8, caspase‐9, and Bid activation in Ewing sarcoma.^51^ Another study demonstrated that melatonin synergized with 5‐fluorouracil (5‐FU) to inhibit cell proliferation and colony formation and promoted the activation of the caspase/PARP‐dependent pathway.^52^ A previous study demonstrated that taurine treatment resulted in a 25‐fold increase in the rate of melatonin production by the pineal gland.^53^ Additionally, taurine exists in developing nervous tissues.^54^ These results imply that drug‐induced neuroblastoma cell differentiation can be used as a potential therapeutic strategy in clinical applications.

Nerve growth factor requires the activation of autophagy. The autophagy process clears out exhausted mitochondria, such as mitochondrial mass.^55^ Additionally, dopaminergic neuron differentiation induces mitochondrial fusion with lysosomes.^56^ A previous study demonstrated that autophagy promotes SH‐SY5Y cell differentiation after treatment with tris(1,3‐dichloro‐2‐propyl) phosphate (TDCIPP), which induces cytoskeletal components and neurite outgrowth.^57^ Melatonin treatment increases the levels of Beclin 1 and counteracts the autophagy‐inhibiting effects of bafilomycin A1, an inhibitor of autophagic flux, indicating that melatonin promotes autophagy.^58^ Another study demonstrated that in the first neurons to differentiate in the retina, the mitophagy receptor BNIP3L/NIX increased. These results indicate that mitochondrial dysfunction may play a prominent role.^59^

In summary, our data demonstrate that physiological concentrations of melatonin can prevent tumors and may be able to eliminate early‐stage neuroblastoma (N2a) cells by upregulating the HAS3 protein and triggering differentiation. This study provides the molecular basis for a novel pathway of neuroblastoma differentiation in which melatonin treatment upregulates HAS3.

## CONFLICT OF INTEREST

All authors have no conflict of interest.
