# Supplementary figures and images for: Melatonin promotes neuroblastoma cell differentiation by activating hyaluronan synthase 3‐induced mitophagy
Source: Cancer Med. 2019 Jul 5;8(10):4821–35. doi: 10.1002/cam4.2389 (PMC6712479; doi:10.1002/cam4.2389)

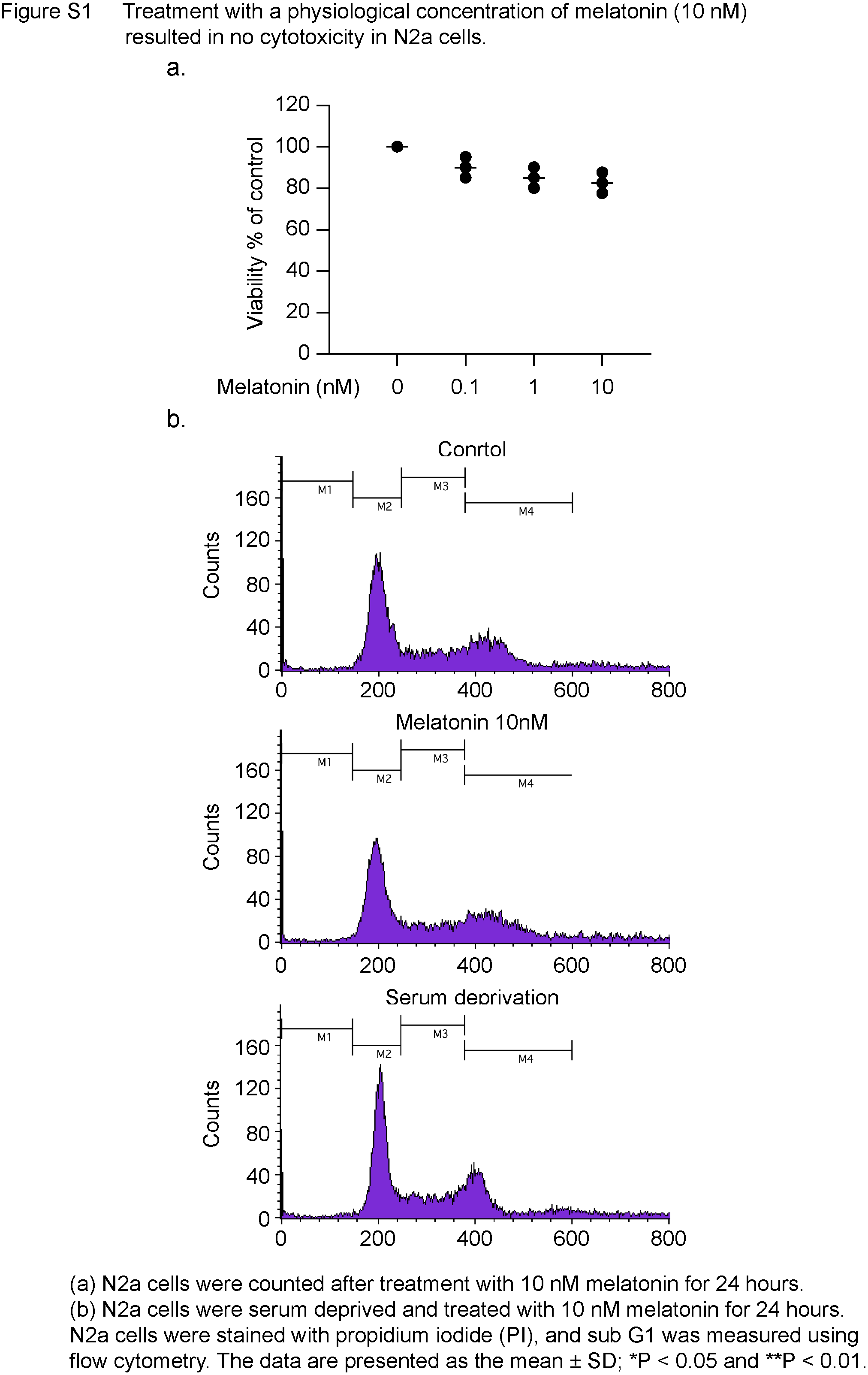

Supplement: Supplementary file 1 [file CAM4-8-4821-s001.tif]

Figure S2 (revised)

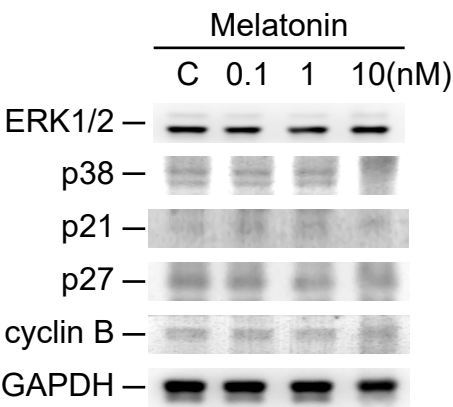

Supplement: Supplementary file 2 [file CAM4-8-4821-s002.pdf]
